# Dipolar cortico-muscular electrical stimulation: a novel method that enhances motor function in both - normal and spinal cord injured mice

**DOI:** 10.1186/1743-0003-7-46

**Published:** 2010-09-17

**Authors:** Zaghloul Ahmed

**Affiliations:** 1 Department of Physical Therapy and Neuroscience Program, The College of Staten Island/CUNY, 2800 Victory Boulevard, Staten Island, NY 10314, USA

## Abstract

**Background:**

Electrical stimulation of the central and peripheral nervous systems is a common tool that is used to improve functional recovery after neuronal injury.

**Methods:**

Here we described a new configuration of electrical stimulation as it was tested in anesthetized control and spinal cord injury (SCI) mice. Constant voltage output was delivered through two electrodes. While the negative voltage output (ranging from -1.8 to -2.6 V) was delivered to the muscle via transverse wire electrodes (diameter, 500 μm) located at opposite ends of the muscle, the positive output (ranging from + 2.4 to +3.2 V) was delivered to the primary motor cortex (M1) (electrode tip, 100 μm). The configuration was named dipolar cortico-muscular stimulation (dCMS) and consisted of 100 pulses (1 ms pulse duration, 1 Hz frequency).

**Results:**

In SCI animals, after dCMS, cortically-elicited muscle contraction improved markedly at the contralateral (456%) and ipsilateral (457%) gastrocnemius muscles. The improvement persisted for the duration of the experiment (60 min). The enhancement of cortically-elicited muscle contraction was accompanied by the reduction of M1 maximal threshold and the potentiation of spinal motoneuronal evoked responses at the contralateral (313%) and ipsilateral (292%) sides of the spinal cord. Moreover, spontaneous activity recorded from single spinal motoneurons was substantially increased contralaterally (121%) and ipsilaterally (54%). Interestingly, spinal motoneuronal responses and muscle twitches evoked by the test stimulation of non-treated M1 (received no dCMS) were significantly enhanced as well. Similar results obtained from normal animals albeit the changes were relatively smaller.

**Conclusion:**

These findings demonstrated that dCMS could improve functionality of corticomotoneuronal pathway and thus it may have therapeutic potential.

## Introduction

After a spinal cord injury (SCI), spared regions of the central nervous system are spontaneously capable of repairing the damaged pathway, although the process is very limited. Moreover, despite the many promising treatment strategies to improve connections across the damaged spinal cord, the strength of connectivity and functional recovery of the impaired spinal cord is still unsatisfactory. It is well known that spared axons sprout after an SCI [1-3], but fine-tuning of this process as well as synapse stabilization might be dependent on precise pathway-selective activity. Electrical stimulation is an effective method that promotes reactive sprouting through which an increase in the number of functional connections may be possible [3]. Electrical stimulation can also improve functional connections by strengthening the weak existing synapses and/or by promoting synaptogenesis. Of relevance, one of the emerging concepts is that the nervous system contains latent pathways that can be awoken by electrical stimulation or pharmacological manipulation [3-8].

The majority of the methods employing electrical stimulation use unipolar or bipolar stimuli delivered locally at one region of the nervous system. The loss of neuromuscular activity after SCI leads to inevitable abnormalities that limit the effectiveness of localized stimulation. Some of these abnormalities are muscle atrophy [9-12] and peripheral nerve inexcitability [13,14]. Furthermore, changes of the sensorimotor pathway below and above the lesion may involve several different mechanisms; some of them may be maladaptative [15-17]. This maladaptive function will bias stimuli toward connections with better integrity, further limiting the effectiveness of localized stimulation.

According to the Habbian plasticity principle [18], physiological processes strengthen synaptic connections when presynaptic activity correlates with postsynaptic firing. This phenomenon is known as long term potentiation (LTP) [19]. LTP could be induced by high-frequency presynaptic stimulation or by pairing low-frequency stimulation with postsynaptic depolarization. LTP can also be induced if a pre-synaptic input is activated concurrently with post-synaptic input [20]. In addition, direct current passed through a neuronal pathway can modulate the excitability of that pathway depending on the current polarity and neuronal geometry [21,22]. In that, anodal stimulation would excite while cathodal stimulation inhibits neuronal activity. Drawing from these principles and findings, it was predicted in the present study that encompassing characteristics of current application like pairing cortical with muscular stimulation combined with polarizing current would initiate physiological processes that strengthen connections of the corticomotoneuronal pathways weakened by SCI.

In the present study, we asked the question whether the passage of pulsed direct current across the corticomotoneuronal pathway promotes stronger connections between spinal motor circuits and the motor cortex. Given the electrodes' location, this configuration was called dipolar cortico-muscular stimulation (dCMS). The positive electrode was situated at the motor cortex and the negative electrode was at the contralateral partially isolated gastrocnemius muscle. Here, it was demonstrated that dCMS substantially improved cortically-elicited muscle contractions and spinal cord responses in control and SCI animals.

## Methods

### Animals

Experiments were carried out on CD-1, male and female adult mice in accordance with NIH guidelines. All protocols were approved by the College of Staten Island IACUC. Animals were housed under a 12 h light-dark cycle with free access to food and water.

### Spinal cord contusion injury

Mice were deeply anaesthetized with ketamine/xylazine (90/10 mg/kg i.p.). A spinal contusion lesion was produced (n = 15 mice) at spinal segment T13 using the MASCIS/NYU impactor [23]. 1 mm-diameter impact head rod (5.6 g) was released from a distance of 6.25 mm onto T13 spinal cord level exposed by a T10 laminectomy. After injury, the overlying muscle and skin was sutured, and the animals were allowed to recover under a heating lamp at 30°C. To prevent infection after the wound was sutured, a layer of ointment contained gentamicin sulfate was applied. Following surgery, animals were maintained under pre-operative conditions for 120 days before testing. The time of recovery was selected to ensure that animals developed a stable chronic spinal cord injury.

### Behavioral testing

Behavioral testing (n = 15 animals with SCI) was performed 120 days post-injury to confirm that animals developed behavioral signs of locomotor abnormalities, spasticity syndrome, and sensorimotor incoordination at the hindlimbs. We have only used animals that demonstrated higher (approximately symmetrical in both hindlimbs) behavioral abnormalities. After acclimatization to the test environment, three different testing procedures were used to quantify these behavioral problems.

#### Basso mouse scale (BMS)

Motor ability of the hindlimbs was assessed by the motor rating of BMS [24]. The rating is as follows: 0, no ankle movement; 1-2, slight or extensive ankle movement; 3, planter placing or dorsal stepping; 4, occasional planter stepping; 5, frequent or consistent planter stepping; no animal scored more than 5. Each mouse was observed for 4 min in an open space, before a score was given.

#### Abnormal pattern scale (APS)

After SCI, animals usually developed muscle tone abnormalities that were exaggerated during locomotion and lifting the animal off the ground (by the tail). We developed APS to quantify the number of muscle tone abnormalities demonstrated by animals after SCI in two situations: on ground and off ground. The rating is as follows: 0, no abnormalities; 1, for each of the following abnormalities: limb crossing of midline, abduction, and extension or flexion of the hip joint, paws curling or fanning, knee flexion or extension, ankle dorsi or planter flexion. The total score is the sum of abnormalities from both hindlimbs. The maximal score in APS is 12. Abnormal patterns were usually accompanied by spasmodic movements of the hindlimbs.

#### Horizontal ladder scale (HLS)

For accurate placing for the hindlimb, animals have to have control coordination between sensory and motor systems. To test for sensorimotor coordination, we used a grid with equal spacing (2.5 cm). Animals were placed on the grid and were allowed to take 20 consecutive steps. Foot slips were counted as errors.

### Electrophysiological procedures

Intact (n = 10) and SCI (n = 21) animals underwent a terminal electrophysiological experiment. Animals were anesthetized using ketamine/xylazine (90/10 mg/kg i.p.), which was found to preserve corticospinal evoked potential [3,25-27]. Electrophysiological procedures started approximately 45 min after the first injection to maintain anesthesia at light to moderate level, as recommended by Zandieh and colleagues [25]. Anesthesia was kept at this level using supplemental dosages (~5% of the original dose).

The entire dorsal side of each animal was shaved. The skin covering the two hindlimbs, lumbar spine, and the skull was removed. Both gastrocnemii muscles were carefully separated from the surrounded tissue preserving blood supply and nerves. The tendon of each of the muscles was threaded with a hook shaped 0-3 surgical silk, which was connected to the force transducers. Next, we performed a laminectomy in the 2^nd^, 3^rd^, and 4^th ^lumbar vertebrae (below the lesion in animals with SCI); the 13^th ^rib was used as a bone land mark to identify the level of spinal column. Since spinal cord levels are ~ 3 level displaced upward relative to vertebral levels, we assumed that recording was performed at spinal cord levels: 5^th ^and 6^th ^lumbar and 1^st ^sacral. A craniotomy was made to expose the primary motor cortex (M1) (usually the right M1) of the hindlimb muscles located between 0 to -1 mm from the Bregma and 0 to 1 mm from midline [28]. The dura was left intact. The exposed motor cortical area was explored with a stimulating electrode to locate the motor point from which the strongest contraction of the contralateral gastrocnemius muscle was obtained using the weakest stimuli. In experiments aimed to test the effect of dCMS on nonstimulated motor pathway, two craniotomies were made over the right and left hind limb areas of M1.

Both hind and fore limbs and the proximal end of the tail were rigidly fixed to the base. Both knees were also fixed into the base to prevent transmitting any movement from stimulated muscles to the body and vice versa. Muscles were attached to force displacement transducers (FT10, Grass Technologies, RI, and USA.) and the muscle length was adjusted to obtain the strongest twitch force (optimal length). The head was fixed in a custom made clamping system. The whole setup was placed on an anti-vibration table (WPI, Sarasota, FL, USA). Animals were kept warm during the experiment with radiant heat.

**Figure 1:**
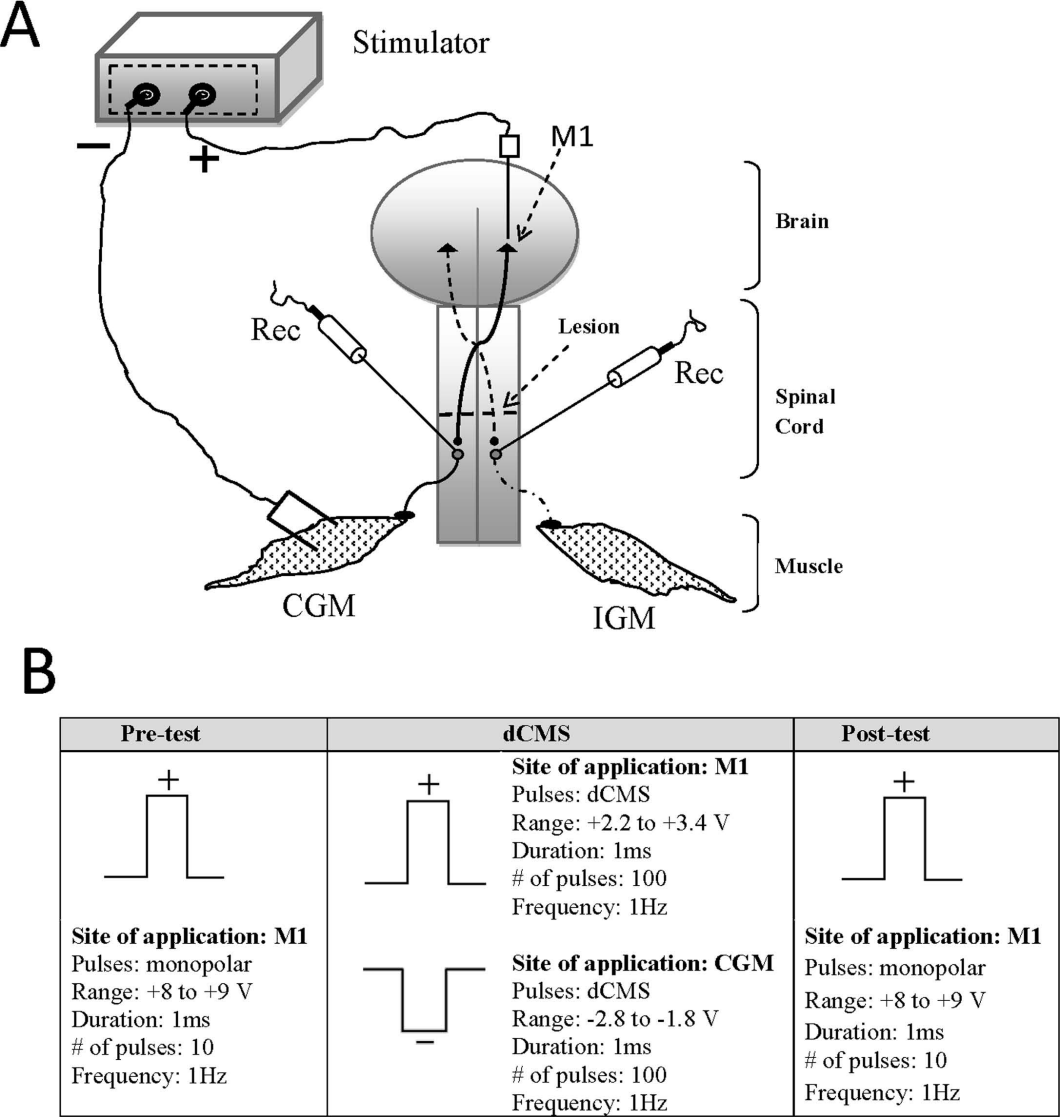
**Experimental setup and procedure**. A: The diagram illustrating the experimental set-up for dipolar cortico-muscular stimulation (dCMS). The positive and negative voltage outputs were connected to electrodes situated on the primary motor cortex (M1), and on the contralateral gastrocnemius muscle, respectively. Both gastrocnemii muscles were attached to force transducers (not shown). Recording from single motoneuron (Rec) was performed simultaneously on each side of the spinal cord below the lesion, as shown. IGM - ipsilateral gastrocnemius muscle, CGM - contralateral gastrocnemius muscle. B: The experimental procedure consisted of three phases designed to stimulate the preparation and to evaluate its reactions to dCMS. The force of muscle contraction and cortically-elicited spinal responses were evaluated before and after the application of dCMS in Pre-test and Post-test phases by application of ten monopolar pulses. The type of stimulation and location of the stimulation and recording electrodes was the same in these two phases. During dCMS phase the preparation was stimulated by application of the positive and negative pulses to the motor cortex (M1) and contralateral gastrocnemius muscle (CGM) respectively. While the number of pulses delivered during Pre- and Post-test phases was the same (10), the number of pulses delivered during dCMS was 100. The duration (1 ms) and the frequency of stimulation (1 Hz) were the same in all three phases of the experiment. The shape of the stimulating current at each phase is shown. There was a continuous recording of ipsilateral and contralateral muscle twitches and evoked and spontaneous spinal activity during the entire experiment.

A stainless steel stimulating electrode (500 μm shaft diameter; 100 μm tip) (FHC, ME, USA) was set on the exposed motor cortex. Paired stainless steel stimulating electrode (~15 mm spacing; 550 μm diameter) was placed on the belly of the gastrocnemius muscle, see Figure 1 (the same electrode was alternated between left and right muscles according to experimental procedure). Electrodes were then connected to stimulator outputs (PowerLab, ADInstruments, Inc, CO, USA). Extracellular recordings were made with pure iridium microelectrodes (0.180 shaft diameter; 1-2 μm tip; 5.0 MΩ) (WPI, Sarasota, FL, USA). Two microelectrodes were inserted through two small openings that were carefully made into the spinal dura matter on left and right sides of the spinal cord. The insertion was made at approximately the same segmental level of the spinal cord. Reference electrodes were placed in the tissue slightly rostral to the recording sites. The ground electrodes were connected to the flap of skin near the abdomen. Motorized micromanipulators (Piezo-translator, WPI, Sarasota, FL, USA) were used to advance the microelectrodes into the ventral horns. The record of extracellular activity was passed through a standard head stage, amplified, (Neuro Amp EX, ADInstruments, Inc, CO, USA) filtered (bandpass, 100 Hz to 5 KHz), digitized at 4 KHz, and stored in the computer for further processing. A power lab data acquisition system and LabChart 7 software (ADInstruments, Inc, CO, USA) were used to acquire and analyze the data.

Once a single motoneuron was isolated at the left and right side of the spinal cord, few antidromic pulses (range, -9 to -10 V) were applied to the homonymous gastrocnemius muscle. As described by Porter [29], the presence of antidromically-evoked response with a short latency (3.45 ms) indicated that the recording electrode was placed in the vicinity of the neuron innervating stimulated muscle. These recordings were also used to calculate the latency of ipsilateral and contralateral spinal responses to muscle stimulation. A cortical pre-test stimulation of 10 pulses (anodal monopolar) at maximal stimulus strength (usually +8 to +10 V) was applied to the primary motor cortex (M1). Maximal stimulus strength was defined as the strength of stimulation when no further increase in muscle contraction was observed. This was also used to calculate the maximal threshold of M1 stimulation.

Next, dCMS was applied through two electrodes as shown in Figure 1. The negative output was connected to an electrode situated on the gastrocnemius muscle and the positive electrode was at M1 (Figure 1). The voltage strength and polarity were computer-controlled (LabChart, ADInstruments, Inc, CO, USA). Different combinations of stimulus parameters were tried before determining the one with the best responses. The strength of dCMS stimulation was adjusted so that contraction of the ipsilateral muscle (to M1) was at maximal strength which was reached just before the appearance of tail contraction (visually observed). This level of response was achieved by simultaneously applying a negative output (range, -2.8 to -1.8 V) to the muscle and positive output (range, +2.2 to +3.2 V) to M1. At this maximal strength, dCMS was delivered (100 pulses, 1 ms pulse duration, 1 Hz frequency), 15 to 20 seconds after the stimulating paradigm was ended, a post-test (with identical parameters as pre-test) stimuli were delivered to M1. See Figure 1B for experimental design. Thereafter, spontaneous activity was followed for 5 min, then the experiment was ended and animals were injected with a lethal overdose of anesthesia. In a subgroup of animals, the maximal threshold of M1 was re-tested. In addition, in this subgroup, in order to determine the duration of dCMS effect, the magnitude of cortically-elicited muscle twitches and spinal responses were retested every 20 min for 60 min after dCMS.

### White matter staining

At the end of each experiment, animals were injected with a lethal dose of Ketamine. Two parts of the spinal column (including vertebrae and spinal cord) were dissected, one part (1.5 cm) included the lesion epicenter and another part (~0.5 cm) included the recording area (to confirm the electrodes location). Tissues were kept overnight (4°C) in 4% paraformaldehyde in 0.1 m PBS and cryoprotected in 20% sucrose in PBS at 4°C for 24 h. The spinal column was freeze mounted and cut into 30 μm sections and placed on poly-L-lysine-coated glass slides. The spinal column part including the lesion epicenter was sequentially sectioned. Slides were numbered to identify their locations relative to the lesion epicenter.

4 slides from each SCI animal (n = 6) containing the lesion epicenter and 2 slides containing no signs of damaged spinal cord tissue from above and below the lesion were taken for luxol fast blue (Sigma) staining. The lesion epicenter was identified as the section containing the least amount of Luxol fast blue. Sections from control animals (n = 3) at spinal cord T13 level were stained with luxol fast blue. Sections from the recording area were stained with cresyl violet.

The amount of spared white matter was measured using Adobe Photoshop CS4 (Adobe Systems, San Jose, CA). To assess the extent of the spinal cord damage we compared the spared white matter at the lesion epicenter with white matter at spinal cord level T13 in control animals.

### Data analysis

To evaluate the latencies, we recorded the time from the start of the stimulus artifact to the onset of the first deflection of spinal response. Measurements were made with a cursor and a time meter on LabChart software. The amplitude of spinal responses was measured as peak-to-peak. Analysis of muscle contractions were performed with peak analysis software (ADInstruments, Inc, CO, USA), as the height of twitch force measured relative to the baseline. Spike Histogram software was used to discriminate and analyze extracellular motoneuronal activity. All data are reported as group means ± standard deviation (SD). Paired student's *t*-test was performed for before-after comparison or two sample student's *t*-test to compare two groups; statistical significance at the 95% confidence level (p < 0.05). To compare responses from both sides of spinal cords recorded from control animals and from animals with SCI, we performed one way ANOVA followed with Solm-Sidak *post hoc *analysis. Statistical analyses were performed using SigmaPlot (SPSS, Chicago, IL), Excel (Microsoft, Redwood, CA), and LabChart software (ADInstruments, Inc, CO, USA).

## Results

### Behavioral assessment

A contusion lesion of the spinal cord resulted in the appearance of signs of spasticity syndrome such as crossing of both limbs and fanning of the paws (compare 2A and 2C). These postural changes were quantified using the abnormal pattern scale (APS). APS showed substantial increase for both on (APS_on _9.8 ± 0.70) and off (APS_off _9.8 ± 0.70) ground conditions. These postural abnormalities were also accompanied by reduction in Basso Mouse Scale (BMS) scores from 9 in control mouse to 1.2 ± 0.47 and 1.0 ± 0.63 for right and left hindlimb in SCI mouse (n = 15), respectively. In addition, the number of errors on a horizontal ladder test was close to maximum (20) for left (19.5 ± 0.50) and right (18.83 ± 1.16) hindlimb. Collectively, these results indicate that spinal cord injury procedure used in the current study was reliable in inducing behavioral signs of the injury. This strengthens the interpretation of our data.

### Anatomical assessment

**Figure 2:**
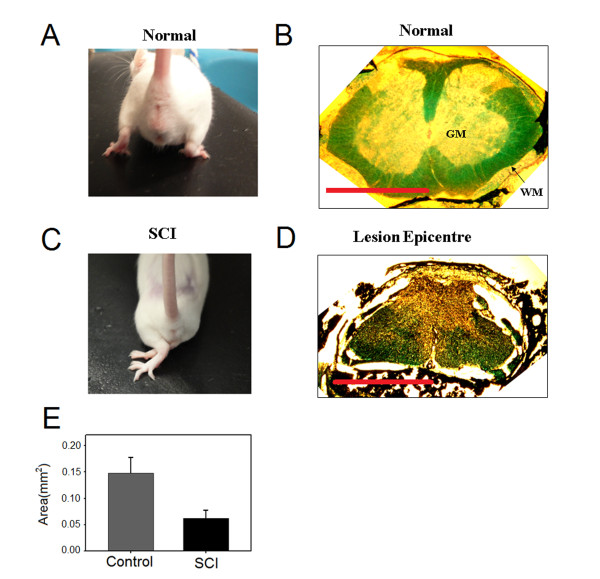
**Anatomical assessment of spinal cord injury**. A: a photograph of control animal shows the control posture of the hindlimbs. B: a representative photograph of spinal cord cross-sectional slice taken from the thoracic level of a control animal. WM - white matter, GM - gray matter. C: a photograph of SCI animal shows the abnormal pattern of the hindlimbs. D: a photograph of a representative slice showing the lesion epicenter taken from SCI animal. Scale bars: 1 mm. E: quantification of spared white matter at the lesion epicenter (n = 6) and from control animals (n = 3). Lesion epicenter had significantly less white matter from control (p = 0.04).

Figure 2 B and 2D show photographs of cross-sectional slices from the thoracic spinal cord region and the lesion epicenter taken from control and SCI animals, respectively. The lesion size was proximally equal in all injured animals tested histologically (n = 6). A rim of white matter was spared on the lateral and ventral side of the spinal cord. The area of spared white matter at the lesion epicenter (0.06 ± 0.03 mm^2^) was significantly reduced 16 weeks after SCI compared to the area of white matter at the same spinal level (0.15 ± 0.06 mm^2^) in control animals (n = 3) (p = 0.04, t-test), Figure 2E. On average, the total cross-sectional area (white and gray matters) of the lesion epicenter was 75 ± 14% of the total cross-sectional area of the same spinal level in control animals.

### Spinal motor neuron identification

**Figure 3:**
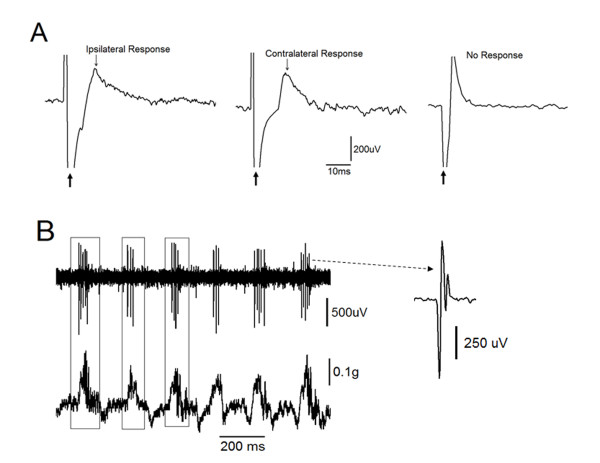
**Identification of spinal motor neurons**. A: responses to the gastrocnemius muscle stimulation. The far left and middle panels show the simultaneous responses of spinal motoneurons located ipsilateral and contralateral to the stimulated gastrocnemius muscle, respectively. The right panel shows recordings from the neuron did not respond to muscle stimulation. B: motoneurons were further identified when their spontaneous activity (upper panel) was time locked with spontaneous contractions at the ipsilateral muscle (lower panel).

Spinal motoneurons innervating the gastrocnemius muscle were at first identified by their large spontaneous spikes. The motoneuronal spike was also accompanied by a distinctive and crisp sound recorded with a loud speaker. Second criterion used to identify spinal motoneurons was their response to the stimulation of the gastrocnemius muscle. Stimulating the gastrocnemius muscle produced a short latency antidromically-generated response that was recorded from motoneurons in the ipsilateral spinal cord. Simultaneously, the microelectrode on the contralateral side of the spinal cord recorded a response that had relatively longer latency than the one picked up from the ipsilateral side. In Figure 3A, three representative conditions were seen during the identification of motoneurons. The left and middle panel show simultaneous motoneuronal responses to stimulated gastrocnemius muscle. The far left panel shows the response of the motoneuron in the ipsilateral side. The middle panel shows the response of the motoneuron in the contralateral side. The far right panel shows a situation when the motoneuron was not responding to the antidromic stimulation of the homonymous gastrocnemius muscle. This confirmed that the unit was not innervating the stimulated gastrocnemius muscle. Third, as depicted in Figure 3B the muscle twitches (lower panel) were correlated with motoneuron activity (upper panel). This association between spontaneous spikes and muscle twitches was used to confirm the connection. In Figure 3B, the enlarged illustration (right) shows typical spike generated by motoneuron. Finally, we histologically confirmed that recording electrodes were localized in the ventral horn of the spinal cord.

### Latencies

**Figure 4:**
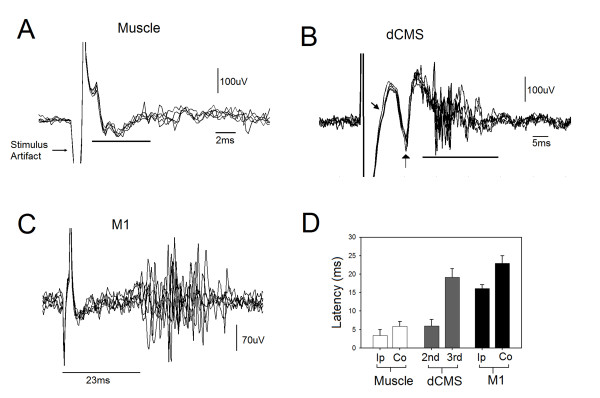
**Spinal responses**. A: six superimposed spinal responses after homonymous gastrocnemius muscle stimulation. The line marks the spinal responses. B: six superimposed spinal responses after dipolar cortico-muscular stimulation (dCMS). C: six superimposed spinal responses after motor cortex (M1) stimulation. The first and second arrows and the line mark the first, second, and third motoneuronal responses to dCMS, respectively, recorded from the contralateral spinal cord to stimulated M1. D: the average latency of spinal responses after muscle stimulation, dCMS (second and third responses), and after M1 stimulation. Ipsilateral spinal response to M1 stimulation (Ip) was significantly faster than the contralateral response (Co) (p < 0.05). Muscle stimulation generated significantly shorter response at ipsilateral motoneuron than the ones at the contralateral side (p < 0.05).

Stimulating the gastrocnemius muscle resulted in short and long latency spinal responses recorded by microelectrodes placed in the ipsilateral and contralateral ventral horns of the spinal cord, respectively. Figure 4A shows superimposed traces of 6 antidromically-evoked responses. While the average latency of antidromically-evoked responses was 3.45 ± 1.54 ms, the average latency of the contralateral responses (not shown) was longer (5.94 ± 1.24 ms) indicating a transynaptic pathway. The difference between ipsilateral and contralateral spinal responses was statistically significant (n = 15, p < 0.001, t-test). Stimulating M1 resulted in ipsilateral and contralateral spinal motoneuronal responses. Figure 4B shows six superimposed contralateral responses. The ipsilateral response is not shown in Figure 4. The average latency of ipsilateral and contralateral responses was 16.09 ± 1.02 ms and 22.98 ± 1.96 ms, respectively. The difference in latency between ipsilateral and contralateral responses (6.9 ms) was statistically significant (n = 15, p < 0.001, t-test). The application of dCMS resulted in successive spinal motoneuronal responses picked up from the contralateral (to M1) electrode. Figure 4C shows six superimposed recorded traces. In this illustration (Figure 4C), three distinctive responses are seen, one with short latency (3.45 ± 1.54 ms), the second with longer latency (6.02 ± 1.72 ms), and a third with much longer latency (19.21 ± 2.28 ms) (n = 15). The latency of the ipsilateral (to M1) spinal motoneuronal responses (not shown) was 6.02 ± 2.8 ms. Figure 4D summaries the average latencies collected during muscle, M1, and dCMS paradigms.

### Changes in cortically-elicited muscle contraction and spinal responses during dipolar cortico-muscular stimulation (dCMS)

**Figure 5:**
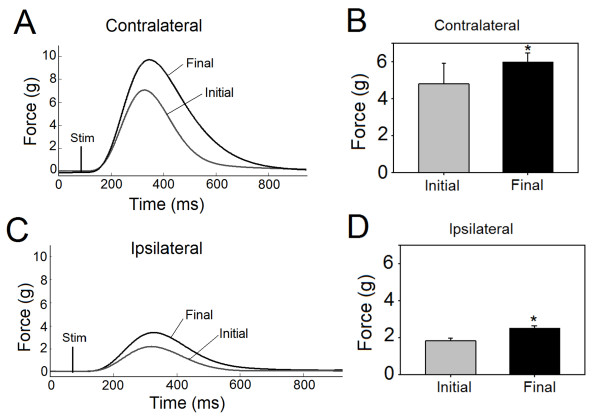
**Muscle contraction during dipolar cortico-muscular stimulation (dCMS) in animals with SCI**. A: representative initial and final muscle twitches demonstrated greater twitch peak force at the end (final) than the beginning (initial) of dCMS on the contralateral muscle to stimulated M1. B: Bars show averages (n = 9) of initial and final twitch peak force of the contralateral muscle, which was significantly larger at the end of dCMS. C: representative initial and final muscle twitches of the ipsilateral muscle (to stimulated M1) during dCMS demonstrated an increase in twitch force in response to dCMS. D: bars show averages (n = 9) of initial and final twitch peak force of the ipsilateral muscle. *p < 0.05. Data show means ± SD.

The application of dCMS gradually increased the twitch peak force recorded from the gastrocnemii muscles and neuronal activity recorded from the spinal cord. Since the magnitude of these enhancements were similar in control and injured animals, only data obtained from SCI animals (n = 9) are presented. The increase in the force of the contralateral cortically-elicited muscle contraction is shown in Figure 5 A&5B. While Figure 5A depicts representative recordings, the averaged results obtained from all 9 SCI animals are shown in Figure 5B. The increase from an initial twitch peak force of 4.8 ± 1.12 g to a final twitch peak force of 6.1 ± 0.71 g was statistically significant (percent change = 25.0 ± 3.8%, p = 0.001, paired t-test). The amplitude of ipsilateral cortically-elicited muscle contraction increased as well. Representative recordings and averaged results are shown in Figure 5C&5D. The final twitch force increased significantly from its initial value of 1.8 ± 0.74 g (percent change = 37.7 ± 1.14%; p = 0.001, paired t-test).

Similar results were obtained by comparing the first and the last spinal motoneuronal responses of the 100 pulses of dCMS protocol. On average, the contralateral (to stimulated M1) spinal motoneuronal responses showed significant increase (percent change = 49.75 ± 16.9%, p = 0.013, one sample t-test), as did the ipsilateral (to stimulated M1) spinal motoneuronal responses (percent change = 48.10 ± 19.8%, p = 0.04, one sample t-test). These findings suggest that physiological processes that mediate stronger connections of the corticomotoneuronal pathway were initiated during dCMS application.

### The influence of dCMS application on cortically-elicited muscle twitches and neuronal activity in SCI animals

**Figure 6:**
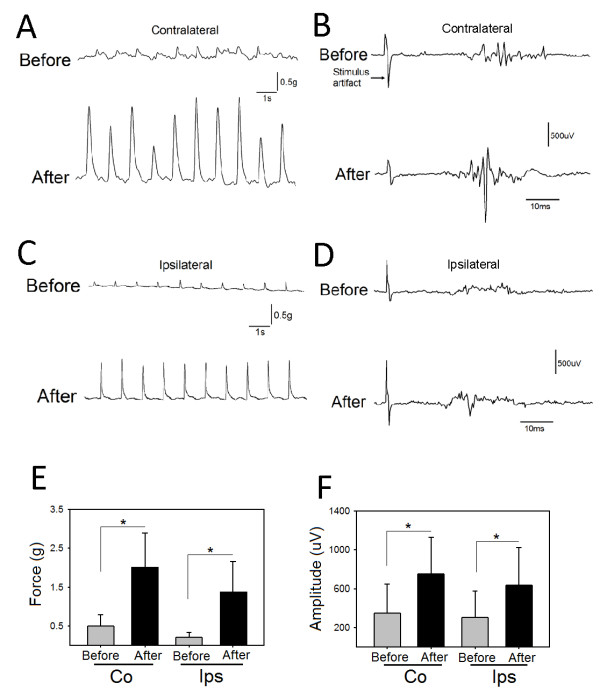
**Dipolar cortico-muscular stimulation (dCMS) augments cortically-elicited muscle contraction and spinal motoneuronal response in animals with SCI**. A: representative gastrocnemius muscle twitches induced by stimulating the contralateral M1, upper and lower panels show muscle twitches before and after dCMS. B: contralateral cortically-elicited spinal response before (upper panel) and after (lower panel) dCMS are shown. C: representative muscle twitches recorded from the ipsilateral (to stimulated M1) gastrocnemius muscle. D: the upper and lower panels show ipsilateral cortically-elicited spinal responses before and after dCMS. E: quantification of results from 9 animals with SCI revealed that contralateral (Co) (to stimulated M1) muscle twitch force was significantly increased, as did the ipsilateral (Ips) (to stimulated M1) muscle twitch force. F: similarly, quantification of cortically-elicited spinal responses from the same animals revealed significant increase in both contralateral and ipsilateral (to stimulated M1) after dCMS. *p < 0.05. Data show means ± SD.

We examined cortically-elicited muscle twitches (measured as peak twitch force) before and after dCMS in SCI animals. In all animals used in these experiments, twitch force was remarkably increased after dCMS. An example of twitches of the contralateral (to stimulated M1) (Figure 6A) and ipsilateral (to stimulated M1) (Figure 6C) gastrocnemius muscles before (upper panels) and after (lower panel) dCMS are shown. We also examined cortically-elicited spinal responses (measured as peak - to - peak), which was also substantially increased. Examples of contralateral (Figure 6B) and ipsilateral (Figure 6D) spinal responses are shown. In Figure 6E, the twitch peak force of the contralateral muscle showed significant increase (n = 9; p < 0.001) (average before = 0.50 ± 0.28 g *vs. *average after = 2.01 ± 0.80 g) (percent change = 456.1 ± 117.5%) after dCMS, as did the twitch peak force of the ipsilateral (to stimulated M1) muscle (average before = 0.21 ± 0.12 *vs. *average after = 1.38 ± 0.77, p < 0.001, paired t-test) (percent change = 457 ± 122.7%). In Figure 6F, spinal motoneuronal responses (n = 9) contralateral (to stimulated M1) showed significant increase after dCMS (average before = 347.67 ± 294.68 μV *vs. *average after = 748.90 ± 380.59 μV, p = 0.027, paired t-test) (increased by 313 ± 197%), as did ipsilateral (to stimulated M1) spinal motoneuronal responses (average before = 307.13 ± 267.27 μV *vs. *average after = 630.52 ± 389.57 μV, p = 0.001, paired t-test) (increased by 292 ± 150%). Data are shown as means ± SD. These results show that dCMS greatly potentiates the corticomotoneuronal pathway in injured animals.

The maximal cortical threshold defined as the lowest electrical stimulus eliciting the strongest muscle twitch peak force was reduced from 9.4 ± 0.89 V to = 5.7 ± 0.95 V after dCMS application (n = 4, p < 0.001, t-test). The cortically-elicited muscle twitch force and the magnitude of spinal motoneuronal responses, evaluated 60 min after dCMS in 5 SCI animals, were still significantly elevated on both sides (p < 0.001).

### Effects of dCMS on the non-stimulated corticomotoneuronal pathway in animals with SCI

The test stimulation of the other M1, contralateral to M1 where dCMS had been applied, revealed an increase of the contraction force recorded from contralateral and ipsilateral gastrocnemii muscles. The increase in contralateral (percent change = 182.8 ± 87.18%), and ipsilateral muscles (percent change = 174.8 ± 138.91%) was statistically significant (n = 6, p < 0.05, t-test).

Contralateral spinal motoneuronal response was increased significantly (p = 0.006, *t-*test) (average percent change = 373.8 ± 304.99%), as did ipsilateral (average percent change = 289.2 ± 289.62%, p = 0.025, *t*-test). These results indicate that even though dCMS was unilaterally applied, it affected the corticomotoneuronal pathway bilaterally.

### The influence of dCMS application on cortically-elicited muscle twitches and neuronal activity in control animals

**Figure 7:**
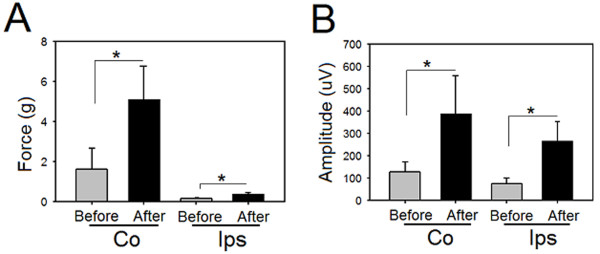
**Cortically-elicited muscle contraction and spinal responses after dipolar cortico-muscular stimulation (dCMS) in control mice**. A: quantification of results from 6 control animals revealed significant increase in contralateral (CO) and ipsilateral (Ips) (to stimulated M1) muscle twitch force after dCMS. B: contralateral (to stimulated M1) cortically-elicited spinal responses were significantly increased after dCMS, as did ipsilateral responses. *p < 0.05. Data show means ± SD.

The application of dCMS across the corticomotoneuronal pathway in control animals (n = 6) resulted in an increase in the cortically-elicited muscle contraction force produced by both gastrocnemii muscles. The twitch peak force of the contralateral muscle increased from 1.62 ± 1.0 g before to 5.12 ± 1.67 after dCMS application (percent change = 250.75 ± 129.35%, p = 0.001, paired t-test, Figure 7A). The twitch peak force of the muscle on the ipsilateral side increased as well, although the increase was less pronounced (from 0.16 ± 0.05 g to 0.39 ± 0.08 g), before and after dCMS, respectively (percent change = 166.38 ± 96.56%, p = 0.001, paired t-test, Figure 7A)

The amplitude of evoked responses recorded from spinal motoneurons was also enhanced by dCMS application. As depicted in Figure 7B, the average amplitude of these spikes recorded at the contralateral side increased from 127.83 ± 46.58 μV to 391.17 ± 168.59 μV (percent change = 168.83 ± 152.00%, p = 0.009, paired t-test). The increase at the ipsilateral side was even greater (percent change = 369.00 ± 474.00%, 77.50 ± 24.73 μV before versus 267.00 ± 86.12 μV after dCMS, p = 0.007, paired t-test).

### Comparison between control and SCI animals

The cortically-elicited muscle twitches of contralateral muscle, recorded from control animals were stronger than twitches observed in SCI animals regardless of whether they were recorded before (p = 0.009, t-test), or after (p = 0.001, t-test) the dCMS procedure. The response of ipsilateral muscles, however, was more complex. Before dCMS, SCI animals showed higher ipsilateral twitch peak force than control animals, although the difference was not statistically significant (p = 0.39, t-test). This difference became statistically significant after dCMS intervention (p = 0.01, t-test).

Similarly, before dCMS, the cortically-elicited responses recorded from spinal motoneurons were higher in SCI animals at ipsilateral and contralateral sides, although the difference did not reach statistical significance (p = 0.13, t-test). However, following dCMS, this difference was increased and became statistically significant (p = 0.009, t-test).

Next we have calculated a relative measure of muscle performance, which we called "fidelity index" (FI). FI is the ratio of cortically-elicited spinal motoneuronal response to the corresponding muscle twitch peak force (spinal response/muscle twitch ratio). Lower fidelity index value indicates better association between spinal responses and their corresponding muscle twitches. In other words, it means better ability of a spinal response to induce muscle contraction. Therefore, changes in this index may indicate changes in relation between spinal and peripheral excitability.

**Figure 8:**
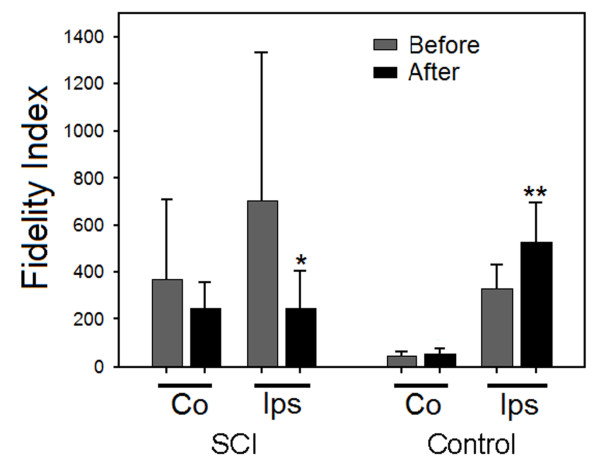
**Fidelity index analysis**. Fidelity index (spinal response/muscle twitch force) was quantified from 6 control and 9 with SCI animals. In animals with SCI, Contralateral (CO) to stimulated M1 fidelity index shows reduction after dCMS but was not statistically significant; however, ipsilateral (Ips) fidelity index was significantly reduced after dCMS. In control animals, after dCMS, Fidelity index was increased contralateral to stimulated M1 but was not statistically significant, however, the ipsilateral Fidelity index was significantly higher after dCMS. Note that the lower the fidelity indexes the better the correlation between muscle contraction and spinal response. *p < 0.05, lower from before dCMS; **p < 0.05, higher from before dCMS. Data show means ± SD.

After dCMS, SCI animals showed overall significant group reduction in FI (F = 3.3, p < 0.033, ANOVA) (Figure 8). Solm-Sidak *post hoc *test showed reduction in contralateral FI (average before = 368.35 ± 342.51 *vs. *average after = 246.15 ± 112.24), however, the difference was not statistically significant (p = 0.46). The ipsilateral FI was significantly reduced after dCMS (average before = 704.59 ± 625.7 *vs. *average after = 247.95 ± 156.27) (p = 0.011). The effect of dCMS treatment was the opposite in control animals which demonstrated overall group increase in FI after this procedure (F = 31.51, p < 0.001, ANOVA). FI was significantly increased after dCMS (Solm-Sidak *post hoc*, p < 0.001) in the ipsilateral side (average before = 328.53 ± 104.83 *vs. *average after 526.83 ± 169.38). There was also a trend reflecting an increase in the contralateral side (average before = 48.59 ± 17.*71 vs. *average after = 56.15 ± 24.19), but was not statistically significant (Solm-Sidak *post hoc*, p = 0.89).

Comparing FI from control animals with FI from SCI animals showed a statistically significant lower index in the contralateral side of control animals (p < 0.001, ANOVA, Solm-Sidak *post hoc*) both before and after dCMS. These results support the findings that peripheral nerves are in-excitable or of higher threshold in subjects with SCI [13].

### dCMS increased spinal motoneurons spontaneous activity

**Figure 9:**
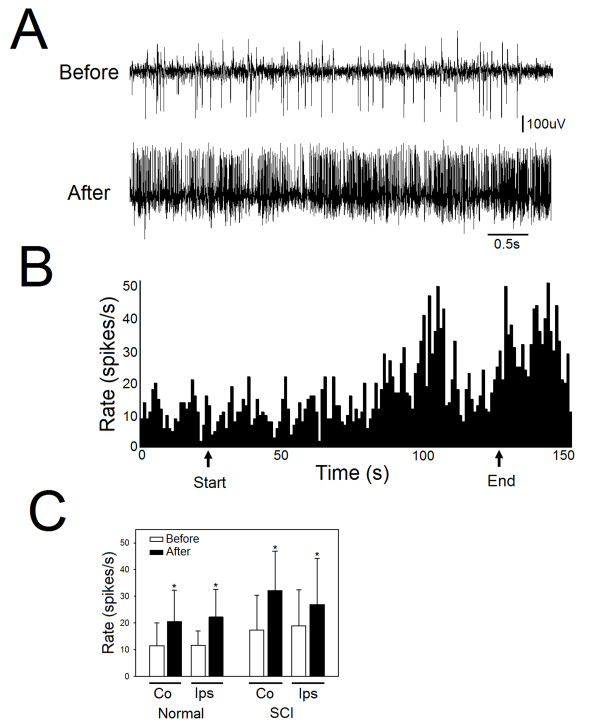
**dCMS increased spontaneous activity of spinal motoneurons (MNs)**. A: an example of spontaneous activity from one MN shows the level of activity before (upper panel) and after (lower panel) dCMS intervention. This example was taken from SCI animal. B: a representative experiment shows firing rate (spikes/s) during an entire experiment. Arrows show the start and end of dCMS application. C: quantification of spontaneous activity before compared with after dCMS show significant increase in both contralateral (Co) and ipsilateral (Ips) spinal recordings from control and with SCI animals. *p < 0.05. Data show means ± SD.

Comparing the firing rate of spontaneous activity before and after dCMS intervention demonstrated significant increase in both control and SCI animals. In Figure 9 A&9B, a representative spontaneous activity recording from an SCI animal is shown. In SCI animals, spontaneous activity was significantly increased in the contralateral side of the spinal cord (average before = 17.31 ± 13.10 spikes/s *vs. *average after = 32.13 ± 14.73 spikes/s; p = 0.001) (121.71 ± 147.35%), as it did in the ipsilateral side (average before = 18.85 ± 13.64 spikes/s *vs. *average after = 26.93 ± 17.25; p = 0.008) (percent change = 54.10 ± 32.29%). In control animals, spontaneous activity was significantly increased in the contralateral (to stimulated M1) side of the spinal cord (average before = 11.40 ± 8.65 spikes/s *vs. *average after = 20.53 ± 11.82 spikes/s; p = 0.006) (percent change = 90.10 ± 42.53%), as it did in the ipsilateral side (average before = 11.63 ± 5.34 spikes/s *vs. *average after = 22.18 ± 10.35 spikes/s; p = 0.01) (percent change = 99.10 ± 1.10%). One way ANOVA showed no significant difference between control and SCI animals in firing rate, although, SCI animals demonstrated higher firing rate.

### Effects of monopolar stimulation of muscle or cortex

In order to determine that the effect was unique to dCMS, the influence of monopolar stimulation (maximal stimulation for100 pulses, 1 Hz frequency) of either the muscle or the motor cortex on spinal motoneuronal response and muscle twitch peak force was examined.

As expected, muscle stimulation resulted in significant reduction in muscle twitch force (-20.28 ± 7.02%, p < 0.001, t-test) (n = 5, 3 SCI and 2 control). It also resulted in a significant reduction in spinal motoneuronal responses evoked by the contralateral (to stimulated muscle) M1 test stimulation (average before = 747.50 ± 142.72 μV, *vs. *average after = 503.14 ± 74.78) (F = 17.11, one way ANOVA, Solm-Sidak *post hoc*, p < 0.001), however, no significant change was seen in responses recorded in the ipsilateral (to stimulated muscle) side of the spinal cord (average before 383.33 ± 140.67 μV *vs. *average after = 371.43 ± 35.61, p = 0.84).

In a separate group of animals (n = 5, 3 SCI and 2 control), we also tested the effect of the monopolar stimulation paradigm applied only at the motor cortex on contralateral muscle twitch peak force and spinal motoneuronal response. Both, the muscle twitch and motoneuron response were significantly reduced by over 50% (-53.69 ± 4.3%, p = 0.001, t-test) and almost 15% (-14.59 ± 9.10%, p = 0.003, t-test), respectively. These results indicate that monopolar muscle or cortical stimulation at maximal strength results in fatigue of muscle twitch force and reduction in spinal responses.

## Discussion

The results show remarkable enhancement of the excitability of the corticomotoneuronal pathway induced by unilateral application of the dCMS. This enhancement was observed in control animals and in SCI animals that had severe locomotor impairment associated with signs of spastic syndrome. The effect was observed both in the ipsilateral and contralateral pathways. The maximal threshold of the ipsilateral cortex was reduced. Improvement in muscle strength was accompanied by an increase in spontaneous activity and potentiation of evoked responses of the spinal motoneurons. The spinal motoneuronal responses and muscle twitches evoked by the stimulation of the contralateral, non-treated M1 were significantly enhanced as well. The dCMS-induced effect persisted beyond the phase of stimulation and extended through the entire period of the experiment (60 min).

Bilateral responses to cortical stimulation have been routinely observed [3,6,30-33]. They can be mediated by interhemispheric connections, ipsilateral cortico-spinal connections (5-6% of the contralateral projections) [34], or commissural spinal neurons. As seen in Figure 6F and 7B, ipsilateral responses to unilateral stimulation of motor cortex evoked larger responses in SCI animals compared to controls. These results further support the idea that ipsilateral corticospinal projections are more efficient in evoking muscle contraction after SCI [3].

The mechanism of the dCMS induced increase in the excitability of the corticomotoneuronal pathway is not clear and one can only speculate as to what processes have been modulated. It is obvious that the potentiation in cortically-elicited muscle contraction during dCMS is not like the potentiation seen after neuromuscular stimulation [35]. While neuromuscular stimulation leads to a brief potentiation of muscle force followed by a steep reduction in force, dCMS leads to a gradually proceeding increase in the amplitude of cortically-elicited muscle contraction. Since the enhancement occurred at contra- and ipsilateral sides, the locus of potentiation is most likely either spinal or supraspinal. The enhancement of cortically-elicited muscle contraction was accompanied by a reduction in maximal threshold to cortical stimulation, an increase in spinal motoneuronal responses, and an increase in cortically-elicited spinal motoneuronal responses. Therefore, one can assume that improvements occurred simultaneously at several functional levels of the corticomotoneuronal pathway.

In view of the fact that the current employed in our stimulation paradigm was always positive at one end and negative at the other, our stimulation can be considered in part polarizing. The paradigm of polarizing current was used to study excitability of different parts of the nervous system [36-39]. In these studies, polarizing current produced potential membrane changes in which hyperpolarization occurs at cellular parts near the positive electrode and depolarization occurs near the negative electrode. Complying with this rule, for example, the situation of two polarizing electrodes on the spinal cord (one on the ventral side and the other on the dorsal side) produced changes in membrane and spike potentials of primary fibers from muscles [36]. In our study we suggest that the current is polarizing during the brief, steady moment of pulse duration (1 ms). Given the electrodes placement, in which negative at the muscle and positive at the cortex, the cell body of corticospinal neurons is expected to hyperpolarize and their nerve terminals depolarize. Moreover, spinal motoneurons expected to hyperpolarize at the cell body and dendrites, and depolarize at the neuromuscular junction. According to cell topography relative to the applied electrical field, membrane potential changes are also expected to occur at intervening interneurons. These membrane changes that occur briefly during each pulse of dCMS, seem to prime corticomotoneuronal pathway for potentiation. In addition, the stimulating pulse has two more periods: rising (0.250 ms) and falling (0.250 ms). These changing periods caused a flow of current that exited from one end and entered at the other end of the corticomotoneuronal pathway. This idea is supported by the observation of stimulus artifact picked up by electrodes in the spinal cord. The current flowed throughout the entire pathway independent from the factors confounding active excitability (see introduction). This might cause activation of the corticomotoneuronal pathway at any possible excitable site/s. This will ensure eliciting *spike-timing-dependent plasticity *[40] that might be one of the mechanisms that mediates the effect of the dCMS. In addition, the high frequency multiple spinal responses, evoked during dCMS, can, in principle, induce *long-term potentiation *[41]. Because dCMS can engage a variety of neuronal mechanisms as well as non-neuronal activity, its effect might be a combination of many changes along the corticomotoneuronal pathway.

The dCMS-induced enhancement of cortically-elicited muscle contraction has been observed in both - control and injured animals. The mechanisms responsible for this amplification in these two groups of animals may overlap, but they do not have to be identical. Although, as discussed above the potentiating effect of dCMS could be mediated by strengthening synaptic responses, the nature and source of these changes may differ substantially in the corticomotoneuronal pathway of control and injured animals. Axonal sprouting is probably the primary source of synaptic connections in the damaged spinal cord [1-3]. However, axonal sprouting does not grant the formation of functional connections. Therefore, one of the probable mechanisms that may mediate the potentiating effect of dCMS is the refining and strengthening of the weak synaptic connections that have resulted from sprouting. Moreover, dormant connections that exist throughout the sensorimotor system [6] may be activated and become functional after dCMS. Potentiating the spared normal connections could also happen after dCMS. On the other hand, in control animals, potentiating normal connections and facilitating dormant connections might be the only processes that mediate the effect of dCMS. The results show that dCMS stimulation was almost twice as effective in injured animals compared with controls. This indicates that injured spinal cord is more prone for dCMS stimulation and posses extra mechanisms mediating the dCMS effect.

In SCI animals, even before the application of dCMS, the spinal motoneurons were responding more aggressively to cortical stimulation than were controls. Nevertheless, very weak or no muscle contraction was seen (Figure 6). This might be due to one of two mechanisms. One would be located in the spinal cord caudal to the lesion and/or the other being, the inexcitable peripheral nerves and/or the irresponsiveness of the muscle. Caudal to the lesion, the activity of the spinal motoneuron pool was probably desynchronized as a result of reorganization. Supporting this idea are the findings by Brus-Ramer and colleagues [3]. The authors reported that chronic stimulation of corticospinal tracts resulted in preferential axonal outgrowth toward the ventral horn. This indicates that inter motoneuronal connections are dynamic processes, which may change by decentralization. Inexcitable peripheral axons were found in patients with SCI [13]. Assuming that the axons in SCI animals are in similar conditions, they could experience an action potential failure resulting in reduced muscle contraction. Muscle atrophy is always seen in animals with SCI [9, 10, and 12] and humans [11]. This might also be one of the reasons why spinal motoneurons responses were not translated adequately into muscle contraction. We quantified the adequacy of motoneuronal responses by calculating the fidelity index, which is the ratio of spinal response to muscle twitch force. The dCMS-induced changes in the fidelity index were opposed in control and injured animals. While this index has been reduced in injured animals, indicating improvement in the effectiveness of the corticomotoneuronal pathway, it had increased in control animals suggesting lowering of the pathway effectiveness probably due to fatigue interference. Therefore, one can imply that injury to the spinal cord initiates processes which favor regeneration of the function. Apparently our procedure synchronizes and facilitates these processes, promoting recovery.

It has been demonstrated that spontaneous activity in spinal motoneurons is a significant factor in developing spinal circuits involved in locomotion [42,43]. It has also been shown that increasing or decreasing the frequency of spontaneous activity will disrupt connectivity in a developing spinal cord [44,45]. In the light of these studies and our data one can ask what role the changes in spinal motoneuronal spontaneous activity play in recovery after SCI, and what are the interactions between interventions, spontaneous activity and functional recovery after SCI? These questions await further investigations which could be guided by our observation that dCMS increased the tonic activity of spinal motoneurons in animals with SCI as well as in control animals. Before the dCMS application, the spontaneous activity of motoneurons in animals with SCI was higher than that of control animals. This and the exaggerated evoked spinal responses in animals with SCI, is consistent with the behavioral measurements that show spastic syndrome-like characteristics. The exaggerated spontaneous firing rate of spinal motoneurons is also consistent with data from motor unit firing in humans and animals after SCI [46,47] and with results from intracellular recordings from sacrocaudal motoneurons that show a sustained and exaggerated firing rate in animals with SCI [48]. Minutes after dCMS, motoneuronal spontaneous activity was still substantially increased. Some of these activities were rhythmic, as shown in Figure 3B, although most of the spontaneous activity was in an un-modulated pattern of firing as shown in Figure 9A. Voltage-dependent persistent inward currents (PICs) that strengthen synaptic inputs in normal behavior depend on descending brain-stem-released serotonin (5-HT) or noradrenalin [49-51]. Here the increase in the spontaneous firing rate and the appearance of modulated activity in some animals after dCMS may indicate better connections with brain-stem centers.

In conclusion, the results showed clear evidence that dCMS is an effective method that enhances the excitability of the corticomotoneuronal connections. This technique has the potential to be used in humans suffering after spinal cord injury, stroke, multiple sclerosis, and others. In practice, it can be employed to strengthen or awaken any weak or dormant pathway in the nervous system.

## Competing interests

Currently applying for a patent relating to the content of the manuscript.
